# A high preoperative serum IL-25 level is a negative prognosis predictor after liver resection for HBV-HCC

**DOI:** 10.3389/fonc.2022.858151

**Published:** 2022-09-02

**Authors:** Shao-hua Chen, Xu Wang

**Affiliations:** ^1^ Department of Hepatobiliary Surgery, 900TH Hospital of Logistics Support Force, Fuzhou, China; ^2^ Outpatient Department, Meng chao Hepatobiliary Hospital of Fujian Medical University, Fuzhou, China

**Keywords:** IL-25, hepatitis B virus, hepatocellular carcinoma, prognosis, biomarker

## Abstract

**Objective:**

The aim of this study was to evaluate the association between preoperative IL-25 levels and HBV-HCC patient outcomes following liver surgery.

**Methods:**

This study enrolled consecutive HCC patients that had undergone liver surgery from 2008 to 2015. Baseline patient clinical properties were assessed to establish predictors of postoperative overall survival and recurrence-free survival (OS and RFS, respectively) following liver resection. In addition, serum IL-25 levels were assessed *via* ELISA.

**Results:**

Cox regression analyses revealed IL-25 levels to be independently related to the OS and RFS of 896 HBV-associated HCC patients. An optimal IL-25 cutoff level of 14.9 μg/ml was identified, with 206 patients in this cohort having IL-25 levels above this threshold. Both the OS and RFS of patients with an IL-25 level <14.9 μg/ml were significantly better after liver resection as compared to those of patients with higher preoperative levels of this cytokine (*p* < 0.05). Cox multivariate regression analyses revealed an IL-25 level ≥ 14.9 μg/L to be an independent predictor of poorer RFS and OS. A combination of IL-25 levels and tumor diameter may be an even more reliable predictor of OS.

**Conclusions:**

IL-25 levels are independent predictors of postoperative survival within HCC patients undergoing liver resection.

## Introduction

Hepatocellular carcinoma (HCC) is one of the most common malignant tumors of the digestive system, with high morbidity and mortality (1, 2). Its early diagnosis is crucial for timely treatment and improvement of survival rate (3). Although ultrasound, magnetic resonance imaging (MRI), and other imaging techniques have greatly improved the accuracy of HCC diagnosis, their application is limited due to their high cost, strong invasiveness, and insensitivity to small tumors (3). Therefore, convenient, inexpensive, noninvasive, and reproducible serum biomarkers have played an important role in the diagnosis of HCC (4). Alpha-fetoprotein (AFP) is a widely used biomarker for the diagnosis of liver cancer, but its diagnostic accuracy is limited because it has a high false-negative rate in the detection of small tumors and early tumors. In addition, AFP may be elevated in some benign liver diseases, such as chronic hepatitis and cirrhosis without HCC (5). At present, the application of AFP in early screening of liver cancer has been controversial (5).

Therefore, it is very important to find new biomarkers related to liver cancer, achieve multi-indicator combined detection, improve the accuracy of early diagnosis of liver cancer, and reduce the rate of missed diagnosis. Over the years, other tumor markers for HCC have been proposed, such as Golgi protein 73 (GP73), Glypican-3 (GPC3), and cytokeratin 19 (CK-19) (6–8). GP73 is considered a potential marker of liver cancer, but serum GP73 levels may also be elevated in patients with liver parenchymal tumors. Therefore, GP73 detection is not suitable for distinguishing HCC from benign liver disease (6). Liu et al. found that serum GPC3 level was increased in patients with liver cancer; however, GPC3 was not sensitive to distinguish benign diseases from early liver cancer (7). Previous studies have shown that CK-19 expression is related to the invasive behavior of HCC, such as low differentiation, metastasis, and microvascular invasion, which indicates that CK-19 can be used as an indicator of survival and recurrence of HCC patients (8). However, these markers have not been considered effective enough for clinical use as indicators for HCC diagnosis.

Chronic inflammation is often a main driver of oncogenesis, and suppressing such inflammation can thus slow or arrest the physiological progression of cancer (9, 10). Inflammatory factors have been closely linked to many solid tumor types, including HCC (11), with certain cytokines including interleukin-6 (IL-6) serving as key mediators of systemic immune responses (12). There have been several previous reports demonstrating that the serum levels of inflammatory factors can predict the development or prognosis of many forms of cancer, including HCC (13). In addition, the understanding of the relationship between IL-25 and clinicopathological features, as well as the role of IL-25 in assessing the diagnostic role in HCC, has not been fully investigated. These findings may contribute to a more complete understanding of the significance of IL-25 in HCC. Here, to resolve these controversies, we measured serum IL-25 levels to evaluate the individual and combined diagnostic performance of IL-25 and AFP for HCC. The diagnostic ability of IL-25 for AFP-negative HCC was also evaluated. In addition, we analyzed the relationship between serum IL-25 levels and clinicopathological features in patients with HCC, in order to investigate the value of IL-25 in assessing the progression and prognosis of HCC. Herein, we therefore explored the prognostic relevance of preoperative IL-25 levels among hepatitis B virus (HBV)-associated HCC cases that had undergone liver resection.

## Patients and methods

### Patients

For this study, HBV-infected patients that had undergone liver transplantation conducted by a single surgical team at the 900th Hospital of Logistics Support Force from January 2008 to June 2015 were retrospectively enrolled. All patients had been diagnosed with HCC as per the European Society for the Study of the Liver (EASL) criteria (14), with pathological examination being used to confirm this diagnosis. Selection criteria for cases that participated in this research cohort were as follows: World Health Organization (WHO) preoperative status = 0–1; Child-Pugh Class A; no macrovascular invasion; no distant metastases; and no preoperative chemotherapy, radiosurgery, radiotherapy, or dermal ethanol injections prior to resection of liver. Patients were HBsAg positive and hepatitis C virus (HCV)-Ab negative. The Hospital Institutional Review Committee of the 900th Hospital of Logistics Support Force confirmed the present research, with cases having given the letter of aware satisfaction.

### Follow-up

For the first 2 years after surgery, patients experienced follow-up every 3 months, and every 6 months thereafter. Hospital staff blinded to study objectives conducted all follow-up. All patients were regularly monitored for recurrence using approaches including AFP analyses, chest x-rays, and abdominal USG, MRI, or CT scans that were conducted every 3 months. HCC recurrence was diagnosed using the same criteria as were used to diagnose the primary disease before surgery. Approaches to treating recurrent diseases included TACE, PRFA, and PEI, with the exact procedure being selected based on patient- and tumor-specific factors.

### Propensity score matching

To diminish the potential for bias inherent in this retrospective analysis, propensity score matching (PSM) was performed. Specifically, cases with low and high IL-25 were matched *via* a PSM approach as described previously (15). Covariates included in this PSM model are Ishak’s inflammation, tumor diameter, AFP, AST, HBeAg, HBV-DNA load, encapsulation of tumor, microvascular invasion, tumor count, and the degree of liver resection. Matching was executed at a 1:1 ratio for cases with low and high IL-25 levels as detailed previously (16).

### ELISA

Levels of serum IL-25 were measured in HCC patient samples with the Human IL-25 DuoSet ELISA kit (R&D Systems) based on the provided directions.

### Statistical studies

All outcomes are given as median (range) or mean ± standard deviation (SD), and were studied by implementing unpaired Student’s *t*-tests or *χ* ^2^ assessments as appropriate. The OS and RFS cases were assessed with Kaplan–Meier curves as well as log-rank measurements. Multivariate and univariate methods were used to guide the design of a prognostic nomogram, which was constructed with the “rms” package using R v.3.5.1 (http://www.r-project.org/). This nomogram was assessed based on measurements of the conformity index (C-index), with rcorrp.cens being used to compare the C-index values for this nomogram to those for other nomograms in Hmisc (17). Analyses of the receiver operating characteristic (ROC) curve were implemented to study nomograms and predictors, with *p* < 0.05 as the threshold of significance.

## Results

### Baseline patient characteristics

**Figure 1:**
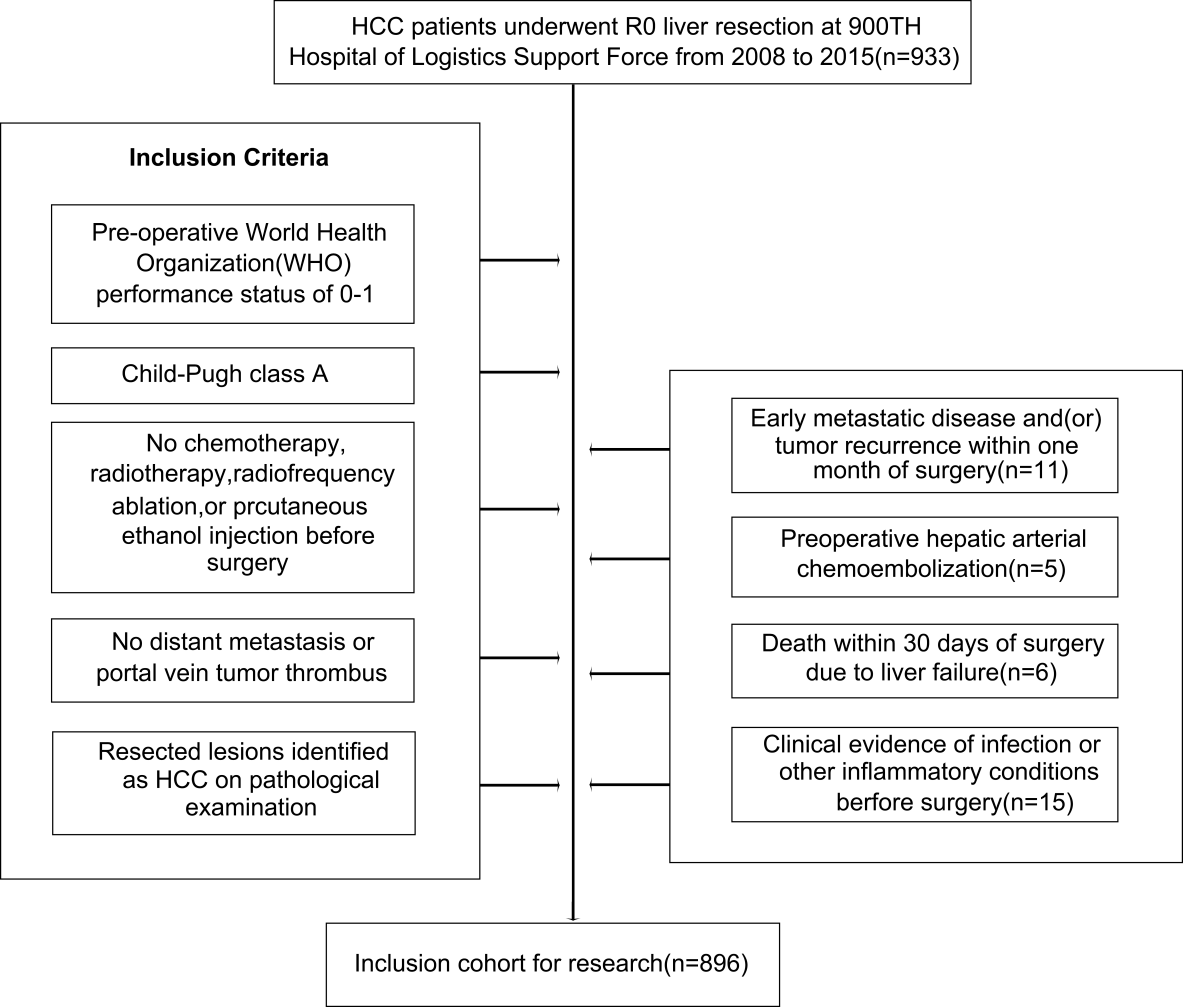
Study population selection.

Over the defined study period, 933 patients with HBV-associated HCC underwent liver transplantation for curative purposes, and were registered in the present survey. Of these cases, 37 were excluded for reasons including early metastasis or recurrence within 30 days postoperatively (*n* = 11), surgery (*n* = 5), liver failure-related mortality within 30 days postoperatively (*n* = 6), or clinically detected preoperative infection (*n* = 15), leaving a cohort of 896 patients eligible for these analyses (**Figure 1**). These patients exhibited a mean age of 52 years (range: 29–75), and were predominantly male (755 male patients and 141 female patients) as shown in **Table 1**. All patients were positive for HBeAg and the remaining 695 were negative for HBeAg. All exhibited Child-Pugh A liver function levels, with a median inflammation level of 6 (range: 2–14). A total of 502 cases exhibited HBV DNA levels ≥ 2,000 IU/ml. Primary tumors were a median of 4.3 cm in size (range: 0.5–17 cm), and serum IL-25 levels ranged from 0.25 to 45 µg/L (median: 10.1 μg/L). Under BCLC staging criteria, 595 patients were stage 0 and 301 were stage B. Of these 896 patients, 325 exhibited microvascular, 421 presented with multiple tumors, and 496 exhibited complete tumor encapsulations. In addition, 130 patients underwent major liver resection. Tumors differed markedly in 145 patients (E-S Grades I and II). The median period of follow-up was 41.5 months (range: 9.5–98.5).

**Table 1:** Patient clinicopathological characteristics.

Characteristics	Total patients (*N* = 896)
Gender	
Male	755
Female	141
Age (years)^a^	52
Liver cirrhosis	
Yes	535
No	361
HBeAg	
Positive	201
Negative	695
AFP (ng/ml)	
≥20	551
<20	345
Alanine aminotransferase (U/L)	
≥40	385
<40	511
Aspartate aminotransferase (U/L)	
≥40	343
<40	553
Total bilirubin (μmol/ml)	
≥17.1	345
<17.1	551
Albumin (g/L)	
≥35	847
<35	49
HBV DNA (IU/ml)	
≥2,000	502
<2,000	394
Ishak inflammation score ^a^	6 (2–14)
Ishak fibrosis score ^a^	4 (1–6)
Tumor diameter (cm)^a^	4.3 (0.5–17)
Tumor encapsulation	
None	400
Complete	496
Major resection	
Yes	130
No	766
Microvascular invasion	
Yes	325
No	571
Tumor number	
Single	475
Multiple	421
Tumor differentiation	
I/II	145
III/IV	751
Stage of BCLC	
0+A	595
B	301

a Age, score of Ishak inflammation, score of Ishak fibrosis, and diameter of tumor are shown as median (range).

HBeAg, hepatitis B e antigen; HBsAg, hepatitis B surface antigen; AFP, alpha-fetoprotein; BCLC stage, Barcelona Clinic Liver Cancer stage.

### IL-25 levels are associated with HCC patient clinicopathological characteristics

**Figure 2:**
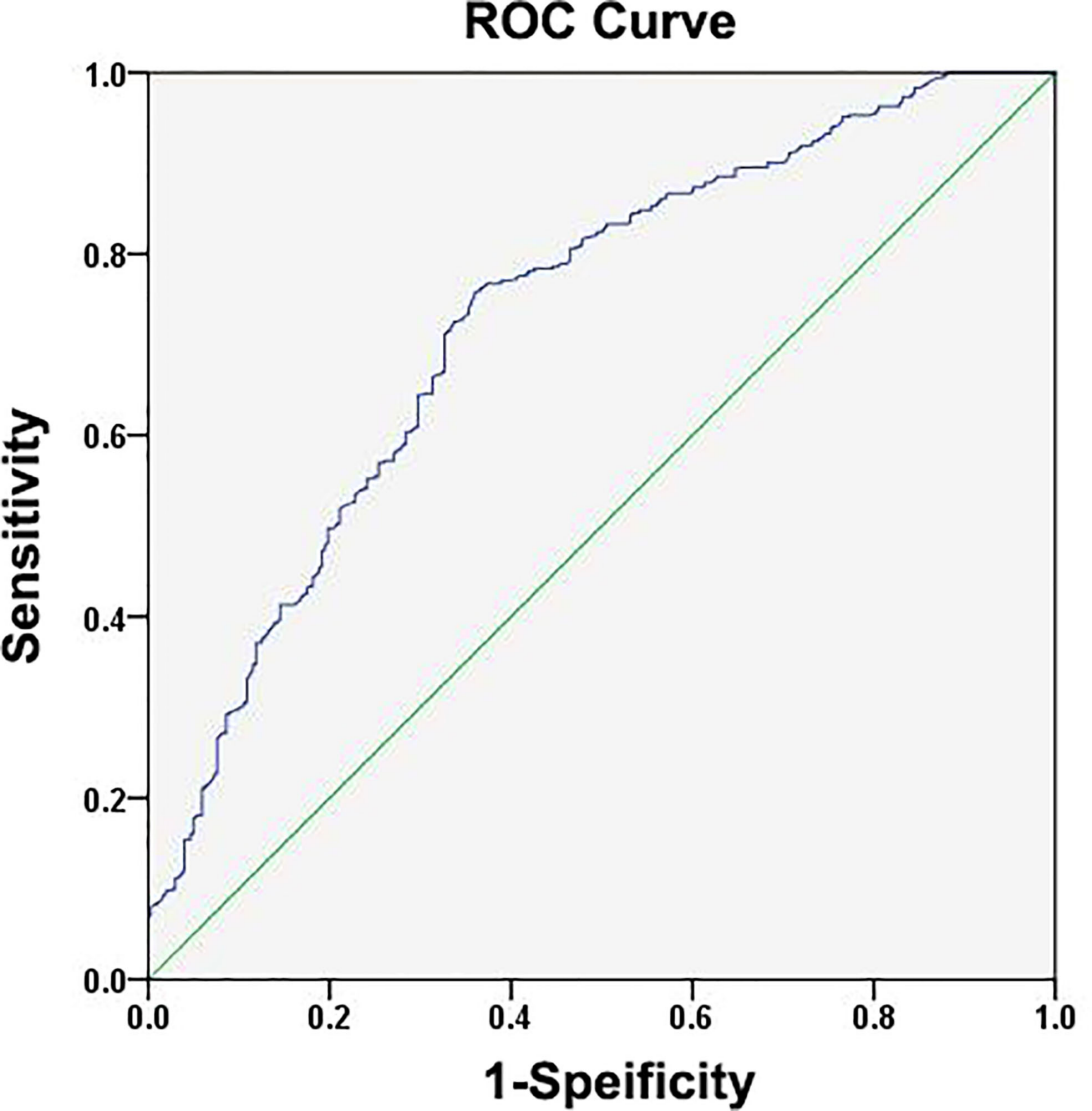
Assessments of the receiver operating characteristic (ROC) curve for the specificity and sensitivity of IL-25 in HCC patients.

Next, ROC curve analyses were used to establish an optimal IL-25 cutoff level capable of differentiating between HCC patient outcomes. The selected cutoff value was 14.9 μg/L, yielding an AUC value of 0.730, a specificity of 0.640, and a sensitivity of 0.757 (**Figure 2**). In total, 334 and 562 patients were respectively clustered into IL-25-high and -low groups, and there were clear differences in clinical characteristics among these groups (**Table 2**). Specifically, individuals with high IL-25 levels exhibited higher AFP levels, greater viral loads (≥2,000 IU/ml), and larger tumor sizes (all *p* < 0.05), indicating that higher IL-25 levels are associated with more advanced HCC. After a PSM analysis, 156 patient pairs were generated (**Table 3**). Following PSM, clinical characteristics did not differ between these cohorts (*p* > 0.05).

**Table 2:** HCC patient clinicopathological and demographic characteristics as a function of IL-25 levels.

	Low IL-25 (<14.9 μg/ml), *N* = 562	Elevated IL-25 (≥14.9 μg/ml), *N* = 334	*p*-value
**Gender** 0.087			
Male	483	272	
Female	79	62	
Age (years)^a^	48.19 ± 10.2	50.23 ± 11.0	0.156
**Liver cirrhosis**			0.778
Yes	338	197	
No	224	137	
**HBeAg**			0.869
Positive	125	76	
Negative	437	258	
**AFP (ng/ml)**			<0.001
≥20	339	212	
<20	223	122	
**Alanine aminotransferase (U/L)**			0.334
≥40	240	145	
<40	322	189	
**Aspartate aminotransferase (U/L)**			0.074
≥40	230	113	
<40	332	221	
**Total bilirubin (μmol/ml)**			
≥17.1	204	141	0.088
<17.1	358	193	
**Albumin (g/L)**			
≥35	535	312	0.288
<35	27	22	
**HBV DNA (IU/ml)**			
≥2,000	255	247	0.000
<2,000	307	87	
Ishak inflammation score^a^	5.12 ± 1.73	5.34 ± 2.98	0.102
Ishak fibrosis score^a^	4.73 ± 1.56	5.08 ± 1.36	0.251
Tumor diameter (cm)^a^	5.47 ± 3.67	8.84 ± 4.14	0.002
**Tumor encapsulation**			0.413
None	249	151	
Complete	313	183	
**Major resection**			
Yes	92	38	0.049
No	470	296	
**Microvascular invasion**			
Yes	193	132	0.131
No	369	202	
**Tumor number**			0.216
Single	289	186	
Multiple	273	148	
**Tumor differentiation**			
I/II	88	57	0.575
III/IV	474	277	

a Age, score of Ishak inflammation, score of Ishak fibrosis, and diameter of tumor are stated as mean ± SD. HbeAg, hepatitis B e antigen; alpha-fetoprotein, AFP.

**Table 3:** HCC patient clinicopathological and demographic characteristics as a function of IL-25 levels after propensity score matching (PSM).

	Low IL-25 (<14.9 μg/ml), *N* = 156	Elevated IL-25 (≥14.9 μg/ml), *N* = 156	*p*-value
Gender			0.732
Male	138	135	
Female	18	21	
Age (years)^a^	50.44 ± 10.59	49.83 ± 10.93	0.522
Liver cirrhosis			0.564
Yes	90	96	
No	66	60	
HBeAg			0.798
Positive	40	43	
Negative	116	113	
AFP (ng/ml)			1.000
≥20	104	104	
<20	52	52	
Alanine aminotransferase (U/L)			1.000
≥40	81	80	
<40	75	76	
Aspartate aminotransferase (U/L)			0.070
≥40	71	88	
<40	85	68	
Total bilirubin (μmol/ml)			0.650
≥17.1	70	75	
<17.1	86	81	
Albumin (g/L)			0.734
≥35	80	77	
<35	76	79	
HBV DNA (IU/ml)			0.650
≥2,000	79	84	
<2,000	77	72	
Ishak inflammation score ^a^	4.93 ± 2.62	5.25 ± 2.68	0.304
Ishak fibrosis score ^a^	4.61 ± 2.99	4.16 ± 2.72	0.138
Tumor diameter (cm)^a^	8.22 ± 4.48	8.31 ± 4.11	0.508
**Tumor encapsulation**			0.908
None	95	93	
Complete	61	63	
**Major resection** Yes	51	46	0.556
No	105	109	
**Microvascular invasion**			0.729
Yes	61	65	
No	95	91	
**Tumor number**			0.4945
Single	73	66	
Multiple	83	90	
**Tumor differentiation**			0.376
I/II	15	21	
III/IV	141	135	

a Age, Ishak inflammation, and diameter of tumor are expressed as mean ± SD. PSM, propensity score matching.

HBeAg, hepatitis B e antigen; alpha-fetoprotein, AFP.

### IL-25 levels are correlated with HCC patient prognosis

**Figure 3:**
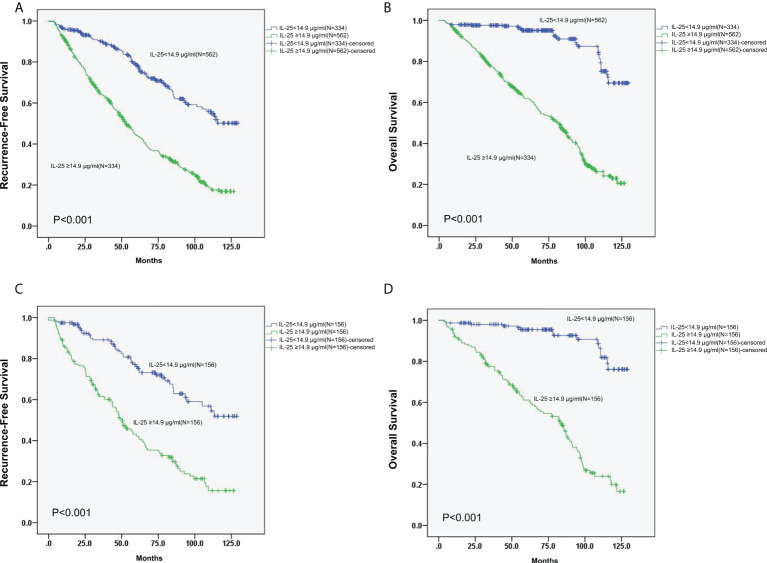
**(A, B)** RFS **(A)** and OS **(B)** curves for all 896 HCC patients with low or high IL-25 levels. **(C)** Curves of RFS for HCC patients in the PSM cohort with low or high IL-25 levels. **(D)** Curves of OS for HCC patients in the PSM cohort with low or high IL-25 levels.

The 3- and 5-year RFS rates of patients in the group of high IL-25 were detected to be considerably decreased in comparison to those of patients in the low IL-25 group (64.1% and 42.2%, respectively, vs. 90.1% and 78.5%, respectively; *p* < 0.05). Higher levels of IL-25 were also associated with decreased 3- and 5-year OS relative to lower IL-25 levels (77.3% and 61.8%, respectively, vs. 97.6% and 95.1%, respectively; *p* < 0.05) (**Figures 3A, B**).

Following PSM, the 3- and 5-year RFS rates in the IL-25-high group were 61.6% and 42.3%, respectively, whereas they were significantly higher at 89.1% and 76.2%, respectively, in the IL-25-low group (*p* < 0.05). Similarly, following the PSM, the 3- and 5-year OS of cases with high IL-25 levels were 77.6% and 61.1%, respectively, which were significantly decreased as compared to those of cases with low IL-25 levels (*p* < 0.05) (**Figures 3C, D**)

### Identification of factors related to HCC patient prognosis

Cox regression analyses were next applied to detect risk factors independently correlated with HCC patient RFS and OS. In univariate analyses, HBV DNA levels, IL-25 levels, AFP levels, microvascular invasion, tumor encapsulation, tumor number, tumor differences, tumor scale, and cirrhosis were all independently correlated with a decreased RFS (**Table 4**), whereas HBV DNA levels, IL-25 levels, AFP levels, tumor encapsulation, and invasion were correlated with worse OS. Subsequently, multivariate approach revealed that HBV DNA levels ≥2,000 IU/ml, IL-25 ≥ 14.9 μg/ml, AFP ≥ 20 ng/ml, a lack of complete encapsulation of tumor, multiple tumors, microvascular invasion, and tumor size ≥ 5 cm were independent predictors of worse patient RFS (**Table 4**), while HBV DNA levels ≥ 2,000 IU/ml, IL-25 ≥ 14.9 μg/L, AFP ≥ 20 ng/ml, a lack of complete tumor encapsulation, and tumor size ≥ 5 cm were independently predictive of worse OS (**Table 5**). Additional analyses of Cox regression were conducted to detect independent predictors of OS and RFS in the cohort of PSM (**Tables 6**, **7**). In this group, an elevated IL-25 level (≥14.9 μg/ml) remained independently associated with poorer RFS and OS.

**Table 4:** Multivariate and univariate studies of factors correlated with HCC patient recurrence-free survival.

	Hazard ratio (95% CI)	*p*-value
**Univariate studies**		
Gender (male vs. female)	0.901 (0.719–1.129)	0.367
Age (years) (≤60 vs. >60)	0.848 (0.695–1.035)	0.105
Alanine aminotransferase (≥40 vs. <40 U/L)	1.212 (1.047–1.302)	0.02
Aspartate aminotransferase (≥40 vs. <40 U/L)	0.504 (0.300–1.741)	0.301
Albumin (<35 vs. ≥35 g/L)	0.806 (0.588–1.105)	0.185
HBV DNA (≥2,000 vs. <2,000 IU/ml)	1.577 (1.564–1.883)	<0.001
Ishak inflammation score (≥3 vs. <3)	1.227 (1.156–1.345)	0.014
Ishak fibrosis score (≥3 vs. <3)	0.825 (0.731–1.267)	0.328
IL-25 (≥14.9 vs. <14.9 μg/L)	1.747 (1.474–2.069)	<0.001
AFP (≥20 vs. <20 ng/ml)	1.649 (1.409–1.929)	<0.001
HBeAg (positive vs. negative)	1.166 (0.995–1.366)	0.058
Tumor encapsulation (yes vs. no)	0.698 (0.603–0.809)	<0.001
Major resection (yes vs. no)	1.168 (0.973–1.403)	0.096
Microvascular invasion (yes vs. no)	1.575 (1.343–1.847)	<0.001
Number of tumor (multiple vs. single)	1.679 (1.377–2.048)	<0.001
Differentiation of tumor (III+IV vs. I+II)	0.560 (0.281–1.899)	0.225
Diameter of tumor (≥5 vs. <5 cm)	1.644 (1.419–1.904)	<0.001
Liver cirrhosis (yes vs. no)	0.056 (0.376–1.442)	0.403
**Multivariate analysis**		
HBV DNA (≥2,000 vs. <2,000 IU/ml)	1.235 (1.133–1.465)	0.013
IL-25 (≥14.9 vs. <14.9 μg/L)	1.494 (1.350–1.786)	<0.001
AFP (≥20 vs. <20 ng/ml)	1.363 (1.254–1.610)	<0.001
Encapsulation of tumor (yes vs. no)	0.785 (0.712–0.879)	0.006
Microvascular invasion (yes vs. no)	1.126 (1.114–1.357)	0.017
Tumor number (multiple vs. single)	1.216 (1.128–1.424)	0.015
Diameter of tumor (≥5 vs. <5 cm)	1.285 (1.188–1.518)	0.003

**Table 5:** Multivariate and univariate studies of factors correlated with HCC patient overall survival.

	Hazard ratio (95% CI)	*p*-value
**Univariate analysis**		
Gender (male vs. female)	0.901 (0.719–1.129)	0.367
Age (years) (≤60 vs. >60)	0.848 (0.695–1.035)	0.105
Alanine aminotransferase (≥40 vs. <40 U/L)	1.212 (1.047–1.302)	0.02
Aspartate aminotransferase (≥40 vs. <40 U/L)	1.504 (1.300–1.741)	<0.001
Albumin (<35 vs. ≥35 g/L)	0.806 (0.588–1.105)	0.18
HBV DNA (≥2,000 vs. <2,000 IU/ml)	1.577 (1.564–1.883)	<0.001
Ishak inflammation score (≥3 vs. <3)	1.227 (1.156–1.345)	0.014
Ishak fibrosis score (≥3 vs. <3)	0.825 (0.731–1.267)	0.328
IL-25 (≥14.9 vs. <14.9 μg/L)	1.747 (1.474–2.069)	<0.001
AFP (≥20 vs. <20 ng/ml)	1.649 (1.409–1.929)	<0.001
HBeAg (positive vs. negative)	1.166 (0.995–1.366)	0.058
Tumor encapsulation (yes vs. no)	0.698 (0.603–0.809)	<0.001
Major resection (yes vs. no)	1.168 (0.973–1.403)	0.096
Microvascular invasion (yes vs. no)	0.575 (0.343–1.847)	0.432
Number of tumor (multiple vs. single)	0.679 (0.377–2.048)	0.501
Differentiation of tumor (III+IV vs. I+II)	0.560 (0.281–1.899)	0.356
Diameter of tumor (≥5 vs. <5 cm)	1.644 (1.419–1.904)	<0.001
Liver cirrhosis (yes vs. no)	1.256 (1.176–1.442)	0.003
**Multivariate analysis**		
HBV DNA (≥2,000 vs. <2,000 IU/ml)	1.235 (1.133–1.465)	0.013
IL-25 (≥14.9 vs. <14.9 μg/L)	1.494 (1.350–1.786)	<0.001
AFP (≥20 vs. <20 ng/ml)	1.363 (1.254–1.610)	<0.001
Tumor encapsulation (yes vs. no)	0.785 (0.712–0.879)	0.006
Diameter of tumor (≥5 vs. <5 cm)	1.285 (1.188–1.518)	0.003

**Table 6:** Multivariate and univariate studies of factors correlated with HCC patient recurrence-free survival in a propensity score matching (PSM) cohort.

	Hazard ratio (95%CI)	*p*-value
**Univariate analysis**		
Gender (male vs. female)	0.619 (0.476–1.039)	0.536
Age (years) (≤60 vs. >60)	0.654 (0.275–0.982)	0.016
Alanine aminotransferase (≥40 U/L vs. <40 U/L)	0.827 (0.382–1.026)	0.569
Aspartate aminotransferase (≥40 U/L vs. <40 U/L)	1.381 (1.167–1.839)	0.026
Albumin (<35 g/L vs. ≥35 g/L)	0.712 (0.369–1.076)	0.876
HBV DNA (≥2,000 IU/ml vs. <2,000 IU/ml)	1.516 (1.084–1.964)	0.001
Total bilirubin (≥17.1 μmol/ml vs. <17.1 μmol/ml)	0.941 (0.672–1.169)	0.532
Score of Ishak inflammation (≥3 vs. <3)	1.519 (1.287 –1.961)	0.021
Score of Ishak fibrosis (≥3 vs. <3)	0.824 (0.384–1.587)	0.198
IL-25 (≥14.9 vs. <14.9 μg/ml)	1.471 (1.028–1.763)	0.001
AFP (≥20 vs. <20 ng/ml)	1.751 (1.537 –2.571)	<0.001
HBeAg (positive vs. negative)	1.473 (1.021–1.694)	0.029
Encapsulation of tumor (yes vs. no)	0.633 (0.632 –1.469)	<0.001
Major resection (yes vs. no)	0.911 (0.681–1.072)	0.723
Microvascular invasion (yes vs. no)	1.629 (1.169 –2.069)	<0.001
Number of tumor (multiple vs. single)	1.957 (1.037 –2.379)	0.001
Differentiation of tumor (III+IV vs. I+II)	0.996 (0.357 –2.467)	0.267
Tumor diameter (≥5 cm vs. <5 cm)	1.051 (0.863–1.714)	<0.001
Liver cirrhosis (yes vs. no)	1.419 (1.167–1.963)	0.036
**Multivariate analysis**		
Age (years) (≤60 vs. >60)	0.993 (0.653–1.279)	0.756
Aspartate aminotransferase (≥40 U/L vs. <40 U/L)	0.914 (0.583–1.327)	0.279
HBV DNA (≥2,000 IU/ml vs. <2,000 IU/ml)	1.469 (1.127–1.937)	0.026
Ishak inflammation score (≥3 vs. <3)	1.716 (1.382–1.973)	0.039
IL-25 (≥14.9 vs. <14.9 μg/ml)	1.487 (1.096–1.672)	0.001
AFP (≥20 vs. <20 ng/ml)	0.961 (0.284–1.037)	0.637
Tumor encapsulation (yes vs. no)	0.758 (0.189–0.836)	0.041
Microvascular invasion (yes vs. no)	0.976 (0.376–1.073)	0.583
Number of tumor (multiple vs. single)	0.993 (0.536–1.376)	0.493
Diameter of tumor (≥5 cm vs. <5 cm)	1.072 (0.753–1.539)	0.001
Differentiation of tumor (III+IV vs. I+II)	0.963 (0.365–1.073)	0.367
Liver cirrhosis (yes vs. no)	1.631 (1.256–1.983)	0.034

Values of HRs (95% CI) and p were determined via multivariate and univariate Cox proportional hazard regression studies.

HbeAg, hepatitis B e antigen; AFP, alpha-fetoprotein; PSM, propensity score matching.

**Table 7:** Multivariate and univariate analyses of factors correlated with HCC patient overall survival in a propensity score matching (PSM) cohort.

	Hazard ratio (95% CI)	*p*-value
**Univariate analysis**		
Gender (male vs. female)	0.756 (0.417–1.391)	0.426
Age (years) (≤60 vs. >60)	0.851 (0.726–1.109)	0.269
Alanine aminotransferase (≥40 U/L vs. <40 U/L)	0.716 (0.541–1.019)	0.654
Aspartate aminotransferase (≥40 U/L vs. <40 U/L)	1.929 (1.172–2.013)	<0.011
Albumin (<35 g/L vs. ≥35 g/L)	0.651 (0.392–1.103)	0.682
HBV DNA (≥2,000 IU/ml vs. <2,000 IU/ml)	1.419 (1.071–1.939)	0.031
Total bilirubin (≥17.1 μmol/ml vs. <17.1 μmol/ml)	0.817 (0.421–1.253)	0.719
Ishak inflammation score (≥3 vs. <3)	1.461 (1.093–1.865)	0.029
Ishak fibrosis score (≥3 vs. <3)	0.910 (0.612–1.382)	0.349
IL-25 (≥14.9 vs. <14.9 μg/ml)	1.563 (1.192–1.829)	0.016
AFP (≥20 vs. <20 ng/ml)	1.792 (1.461–2.031)	<0.001
HBeAg (positive vs. negative)	1.521 (1.069–2.392)	0.042
Tumor encapsulation (yes vs. no)	0.669 (0.479–0.816)	0.011
Major resection (yes vs. no)	0.719 (0.368–1.310)	0.623
Microvascular invasion (yes vs. no)	1.536 (1.217–1.973)	<0.021
Tumor number (multiple vs. single)	1.604 (1.359–2.679)	<0.041
Tumor differentiation (III+IV vs. I+II)	0.593 (0.431–1.618)	0.161
Tumor diameter (≥5 cm vs. <5 cm)	1.329 (1.195–1.921)	<0.001
Liver cirrhosis (yes vs. no)	0.792 (0.538–1.139)	0.435
**Multivariate analysis**		
Aspartate aminotransferase (≥40 U/L vs. <40 U/L)	0.651 (0.493–1.079)	0.791
HBV DNA (≥2,000 IU/ml vs. <2,000 IU/ml)	1.3219 (1.167–1.736)	0.031
Ishak inflammation score (≥3 vs. <3)	0.563 (0.352–1.057)	0.259
IL-25 (≥14.9 vs. <14.9 μg/ml)	1.526 (1.056–1.983)	0.049
AFP (≥20 vs. <20 ng/ml)	0.756 (0.328–1.569)	0.129
HBeAg (positive vs. negative)	0.538 (0.393–1.289)	0.563
Tumor encapsulation (yes vs. no)	0.726 (0.357–1.089)	0.369
Microvascular invasion (yes vs. no)	0.574 (0.346–1.147)	0.573
Tumor number (multiple vs. single)	0.692 (0.379–1.639)	0.134
Tumor diameter (≥5 cm vs. <5 cm)	1.328 (1.125–2.537)	<0.011

The values of HRs (95% CI) and p were determined via multivariate and univariate Cox proportional hazard regression analyses.

HBeAg, hepatitis B e antigen; AFP, alpha-fetoprotein; PSM, propensity score matching.

### Construction and evaluation of nomograms capable of predicting HCC patient survival outcomes

**Figure 4:**
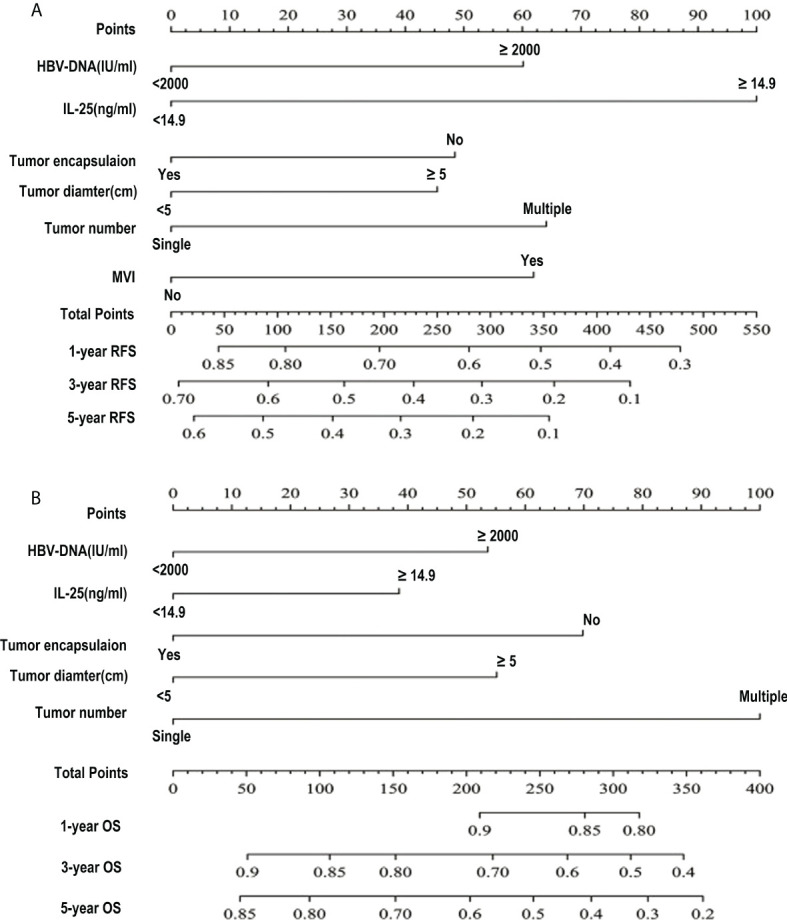
HCC patient survival nomogram. **(A, B)** A survival nomogram designed to assess HCC patient RFS **(A)** and OS **(B)**.

Next, the independent predictors identified in the above multivariate analysis were used to construct nomograms capable of predicting the RFS (**Figure 4A**) and OS of HCC patients (**Figure 4B**). The respective values of the C-index for these nomograms of OS and RFS were 0.726 and 0.645. To explore the predictive value of these nomograms, they were next compared with other independent predictors identified above (**Table 8**). The RFS nomogram C-index value (0.645) was higher than that for HBV DNA (0.542), AFP (0.564), tumor count (0.538), encapsulation of tumor (0.561), IL-25 (0.549), tumor diameter (0.582), and microvascular invasion (0.559) (all *p* < 0.001). The nomogram C-index value (0.726) for OS was higher than that for IL-25 (0.559), HBV-DNA (0.569), AFP (0.584), encapsulation of tumor (0.591), and diameter of tumor (0.635) (all *p* < 0.05). These results thus supported the predictive accuracy of these nomograms, with both the RFS and OS nomograms exhibiting AUC values higher than those for other prognostic risk factors (**Table 9**).

**Table 8:** C-index values for predictors of HCC patient survival outcomes.

	RFS				OS			
Variables	C-index	95% CI	*p* ^†^-value	*p* ^‡^-value	C-index	95% CI	*p* ^†^-value	*p* ^‡^-value
Nomogram	0.645	0.605–0.678		<0.001	0.726	0.675–0.756		<0.001
IL-25	0.549	0.534–0.564	<0.001		0.559	0.538–0.579	<0.001	
AFP					0.584	0.558–0.609	<0.001	0.018
Tumor encapsulation	0.561	0.542–0.580	<0.001	0.076	0.591	0.565–0.618	<0.001	<0.001
Tumor diameter	0.582	0.563–0.602	<0.001	0.599	0.635	0.609–0.661	<0.001	<0.001
HBV-DNA	0.542	0.523–0.562	<0.001	0.064	0.569	0.543–0.595	<0.001	0.413
Tumor number	0.538	0.524–0.549	<0.001	0.005				
Microvascular invasion	0.559	0.543–0.575	<0.001	0.323				

AFP, alpha-fetoprotein.

p^†^-value: nomogram vs. other predictors.

p^‡^-value: IL-25 vs. other predictors.

**Table 9:** ROC curve results pertaining to analyses of the predictors of recurrence-free and overall HCC patient survival.

		RFS			OS			
Variables	AUC	95% CI	*p* ^†^-value	*p* ^‡^-value	AUC	95% CI	*p* ^†^-value	*p* ^‡^-value
Nomogram	0.615	0.602-0.668		0.006	0.653	0.621–0.723		<0.001
IL-25	0.561	0.539–0.583	0.004		0.559	0.533–0.585	<0.001	
AFP					0.590	0.562–0.618	0.003	0.105
Tumor encapsulation	0.566	0.537–0.595	0.001	0.787	0.599	0.570–0.629	0.001	0.026
Tumor diameter	0.568	0.539–0.597	<0.001	0.649	0.624	0.595–0.653	0.001	<0.001
HBV-DNA	0.567	0.538–0.596	0.032	0.723	0.584	0.555–0.613	<0.001	0.227
Tumor number	0.532	0.513–0.551	<0.001	0.048				
Microvascular invasion	0.571	0.547–0.595	0.019	0.547				
**Combination**								
IL-25	0.561	0.539–0.583	<0.001		0.559	0.533–0.585	<0.001	
IL-25+AFP					0.618	0.587–0.648	0.067	0.007
IL-25+Tumor encapsulation	0.496	0.466–0.526	<0.001	0.005	0.463	0.432–0.495	<0.001	<0.001
IL-25+Tumor diameter	0.593	0.563–0.622	<0.001	0.006	0.637	0.606–0.668	0.012	<0.001
IL-25+HBV-DNA	0.603	0.574–0.632	0.019	0.001	0.616	0.586–0.646	0.020	0.007
IL-25+Tumor number	0.574	0.548–0.599	0.021	0.128				
IL-25+Microvascular invasion	0.605	0.578–0.633	0.019	0.026				

AFP, alpha-fetoprotein.

p^†^-value: nomogram vs. other predictors.

p^‡^-value: IL-25 vs. other predictors.

### Assessment of the prognostic value of IL-25 as a predictor of HCC patient survival

For RFS, the C-index value for IL-25 was 0.549, which was considerably greater as compared to that associated with the number of tumors (*p* < 0.05) (**Table 8**). In a ROC curve analysis for RFS (**Table 9**), no differences were observed. The AUC value for IL-25 was higher than that for all other predictors with the exception of tumor number (*p* < 0.05), and in an analysis of multivariate for predictors associated with patient RFS, the HR for IL-25 was the greatest. As IL-25 exhibited the greatest specific weight of any factor in a predictive nomogram for HCC patient RFS, this suggested that IL-25 is the most robust predictor of RFS in this patient population (**Figure 4A**). The C-index value for IL-25 when used to predict HCC patient OS was 0.559, which was the lowest of all tested predictors (**Table 8**) in an analysis of the curve of ROC (**Table 9**). Furthermore, IL-25 exhibited a lower AUC value than any other predictor analyzed in this study, and consistently possessed the least specific weight in a nomogram used to predict patient OS (**Figure 4B**). As such, we evaluated combinations of IL-25 and other predictors with the goal of defining the most reliable prognostic combination associated with patient OS (**Table 9**), revealing that a combination of IL-25 and tumor diameter yielded a greater AUC value than any other combination, thus suggesting that these two parameters may represent a more reliable means of predicting HCC patient OS.

## Discussion

The onset and progression of HCC are driven in large part by interactions between nascent tumor cells and the surrounding inflammatory milieu (18–22). There is thus clear value in further elucidating the specific roles played by particular inflammatory mediators during the progression of cancer (9, 10). HCC is a common malignant tumor of the digestive system, characterized by aggressive growth and early metastasis, and is the second leading cause of cancer mortality in China (2, 3). Because the early symptoms are not obvious, many patients with liver cancer are diagnosed as advanced stage (23). Systematic screening of high-risk groups is necessary for early diagnosis. AFP is the most commonly used biomarker for HCC patients, although its sensitivity and specificity are unsatisfactory, especially for early-stage disease (14, 15). Previous studies have shown that the ability of AFP to diagnose liver cancer is relatively poor. Using cutoff values of 17.76 ng/ml and 21.47 ng/ml would result in 60 (35.71%) and 62 (36.90%) of 168 HCC patients being considered negative. Fifteen of 153 healthy controls (9.80%) and 23 of 150 patients with benign liver disease (15.33%) were considered positive, and these inaccuracies supported the inadequacy of AFP as a biomarker (24). Therefore, new and reliable biomarkers are needed to improve the diagnostic level of liver cancer.

IL-25 is an inflammatory IL-17 family cytokine that is best studied as a driver of type 2 immune responses (13, 25–27). In previous reports, IL-25 was shown to perform a central task in the incidence of acute hepatitis (AH), liver fibrosis, and cirrhosis (28–30). As an anti-inflammatory cytokine, IL-25 promotes type 2 cytokine-dependent immunity and limits the production of pro-inflammatory cytokines by inhibiting the expression of type 1 cytokines. Deregulation of IL-25 has been found in many inflammation-related diseases, including helminth parasite infection, inflammatory bowel disease, asthma, severe hepatitis, and NAFLD (31–33). Meanwhile, IL-25 also plays an important role in several human cancers (31–35). However, it is not completely clear whether IL-25 affects the development of HCC. Studies have shown that IL-25 plays a direct role in cancer cells and affects the development of breast cancer (32–34). Previous results showed that IL-25 did not directly affect the growth, apoptosis, or migration of HCC cells. IL-25-induced M2 macrophages attenuated obesity and NAFLD (36). Similarly, Wang et al. reported that IL-25 induces hepatic macrophages to have an M2 phenotype, negatively regulates the pro-inflammatory immune microenvironment, and improves HDF-induced hepatic steatosis (37). Rizzo et al. reported that IL-25-induced alternatively activated macrophages inhibit colitis (38). In addition, Zhujun Jiang et al. reported that inhibition of IL-25 led to a decrease in the incidence rate of type 2 diabetes  T cells and macrophages in the primary tumor microenvironment, as well as enhanced breast tumor invasion and subsequent lung metastasis (31). These findings suggest that macrophages are the key targets of IL-25, and the activation of M2 phenotype may be the main pathway by which IL-25 promotes the development of HCC. Herein, we found that elevated preoperative IL-25 levels were correlated with features of more advanced HCC and with poorer clinical outcomes (RFS and OS) within HBV-associated HCC cases following the resection of the liver. Tumor recurrence differed significantly between cases with low and high levels of serum IL-25 determined *via* a multivariate analysis approach, with elevated preoperative IL-25 levels being independent predictors of decreased OS and RFS in these cases. Importantly, high IL-25 levels functioned as an accurate predictor of long-lasting survival in cases with early-stage disease. While IL-25 levels were the best-identified predictor of RFS in this study, a combination of IL-25 levels and tumor diameter was better able to predict HBV-associated HCC patient OS. The mechanisms behind these effects are not fully understood, but some researchers believe that IL-25-induced dysregulation of intestinal microbiota promotes hepatocellular carcinoma through alternate activation of macrophages in the tumor microenvironment. Together, these results provide clear evidence that preoperative serum IL-25 levels can predict HCC patient prognosis.

The primary limitation of this research is that it was a single-center retrospective analysis, and it is thus susceptible to potential bias with respect to patient selection. Future large-scale multi-center studies validating and expanding upon our results will thus be essential to affirm the clinical relevance of serum IL-25 as a prognostic biomarker in HBV-HCC patients.

## Conclusion

This study suggests that serum IL-25 levels may be an independent and useful tumor marker for the diagnosis of liver cancer. IL-25 is still valuable in the diagnosis of AFP-negative HCC and can be used as a supplement to AFP in the diagnosis of HCC. The combined diagnosis of the two markers greatly improves the early diagnostic accuracy of HCC. In addition, IL-25 values are associated with several pathological features that represent tumor aggressiveness and/or poor prognosis. Finally, IL-25 could help in the customized management of cases with risk factors for HCC recurrence after liver resection.

## Data availability statement

The original contributions presented in the study are included in the article/supplementary material. Further inquiries can be directed to the corresponding author.

## Ethics statement

The studies involving human participants were reviewed and approved by Ethics Committee of 900TH Hospital of Logistics Support Force (LLH-20150801). The patients/participants provided their written informed consent to participate in this study.

## Author contributions

Conception and design: S-hC and XW; Administrative support: S-hC and XW; Provision of study materials or patients: S-hC and XW; Collection and assembly of data: All authors; Data analysis and interpretation: All authors; Manuscript writing: All authors; Final approval of manuscript: All authors.

## Conflict of interest

The authors declare that the research was conducted in the absence of any commercial or financial relationships that could be construed as a potential conflict of interest.

## Publisher’s note

All claims expressed in this article are solely those of the authors and do not necessarily represent those of their affiliated organizations, or those of the publisher, the editors and the reviewers. Any product that may be evaluated in this article, or claim that may be made by its manufacturer, is not guaranteed or endorsed by the publisher.
